# Daily variation in post traumatic stress symptoms in individuals with and without probable post traumatic stress disorder

**DOI:** 10.1186/s12888-019-2041-7

**Published:** 2019-02-04

**Authors:** Quinn M. Biggs, Robert J. Ursano, Jing Wang, David S. Krantz, Russell B. Carr, Gary H. Wynn, Deborah Probe Adams, Nicole M. Dacuyan, Carol S. Fullerton

**Affiliations:** 1 Center for the Study of Traumatic Stress, Department of Psychiatry, Uniformed Services University of the Health Sciences, 4301 Jones Bridge Road, Bethesda, MD 20814 USA; 2 Walter Reed National Military Medical Center (WRNMMC), Bethesda, MD USA

**Keywords:** Post traumatic stress disorder, Symptom assessment, Ecological momentary assessment, Military personnel

## Abstract

**Background:**

Little is known about the extent to which post traumatic stress symptoms (PTSS) vary from day to day in individuals with post traumatic stress disorder (PTSD). This study examined the variation of PTSS by day of the week, and whether daily or day of week variation differs between individuals with and without probable PTSD.

**Methods:**

Subjects (*N* = 80) were assessed for probable PTSD at enrollment. Using an ecological momentary assessment methodology, PTSS were assessed four times daily by self-report for 15 days. Linear mixed models were used to assess the relationship of PTSS and day of the week.

**Results:**

PTSS varied across the seven days of the week among participants with PTSD (*p* = .007) but not among those without PTSD (*p* = .559). Among those with PTSD, PTSS were lowest on Saturday. PTSS were higher on weekdays (Monday through Friday) versus weekends (Saturday and Sunday) in those with PTSD (*p* = .001) but there were no weekday/weekend differences among those without PTSD (*p* = .144). These variations were not explained by sleep medication, caffeine or alcohol use.

**Conclusions:**

Among individuals with probable PTSD, post traumatic stress symptoms vary by the day of the week, with more symptoms on weekdays compared to weekends. Determination of the factors associated with the daily variation in PTSD symptoms may be important for further developing treatments for PTSD.

## Background

Post traumatic stress symptoms (PTSS) often develop after exposure to a traumatic event, and are associated with clinically significant distress and impairment [1]. PTSS include intrusive re-experiencing of the traumatic event, persistent avoidance of stimuli associated with the event, negative alterations in cognitions and mood, and marked alterations in arousal and reactivity. When PTSS are sufficiently high in number, severity, and duration, they are key elements of the diagnostic criteria for post traumatic stress disorder (PTSD).

Although clinical diagnostic criteria for PTSD are based in part on the presence or absence of PTSS in a particular individual, little is known about how and whether PTSS vary on a day-to-day basis in individuals with and without probable PTSD. The presence of systematic daily variations in PTSS may have broad implications for the assessment and treatment of PTSD. Daily assessment of PTSS may improve the information available for diagnosis, identification of potentially modifiable symptom triggers (e.g., reminders of traumatic events in specific situations and environments), and inform the type and timing of treatment interventions.

Ecological momentary assessment (EMA) is method of repeated experience sampling of subjects in their natural environment that is particularly well-suited to assessing symptom change over time [2–4]. EMA methods have been used to examine a variety of phenomena including anxiety [5], mood [6], eating behavior [7], pain [8], and smoking [9] among others. Studies have examined the association of changes in daily health conditions and behaviors with PTSS including anxiety [10], affect [11], emotion regulation [12], traumatic injury [13, 14], alcohol use [15, 16], smoking [17, 18], and high-risk sexual behavior [19]. However, the specific variation of PTSS by day of week has not been examined.

The purpose of the present study is to determine whether post traumatic symptoms vary by day of the week, and whether there is a relationship between PTSS and the day of the week in military service members with and without probable PTSD. We used EMA methods to determine the day to day and weekday vs. weekend differences in PTSS in subjects during their typical daily routines. PTSS were measured four times a day for 15 consecutive days, which allowed us to examine temporal patterns of PTSS and the extent to which these symptoms differ between weekdays, when most individuals are in their work environments and work demands are present, and weekends, when many of the demands of the work week are usually lessened. We hypothesized that because daily demands would be higher and being in the military work environment may increase exposure to trauma reminders (e.g., weapons, training exercises, combat-related sights, sounds, and experiences) that may trigger PTSD symptoms, PTSS would be higher on weekdays versus weekends in those with probable PTSD but not among those without PTSD.

## Method

### Participants

Current and former U.S. service members (*N* = 98) were recruited from the behavioral health clinic at a large military medical center. A total of *N* = 178 service members were screened, *n* = 111 screened in and of those *n* = 5 declined enrollment and *n* = 8 did not return for the enrollment appointment. Of the *N* = 98 who enrolled, *n* = 80 were included in analyses, *n* = 11 did not return the daily assessments, *n* = 5 did not provide four or more daily assessments, *n* = 1 did not complete the assessment of probable PTSD in the pre-questionnaire, and *n* = 1 was removed as an outlier due to extremely high PTSS scores but failure to meet probable PTSD diagnostic criteria (analyses were conducted with and without the outlier and the conclusions/findings were the same). Participation was voluntary and written informed consent was obtained after the procedures were explained. The study was approved by the Institutional Review Board of Walter Reed National Military Medical Center and the Uniformed Services University of the Health Sciences in Bethesda, Maryland. Data used in this study are part of a larger data collection project looking at post traumatic stress in the U.S. military. Data collection for the larger project continues and studies investigating aspects of post traumatic stress other than day of the week variation in PTSS are planned.

### Procedure and measures

#### Recruitment and enrollment screening

Recruitment was based on self-referral from advertisements or approach to a recruiting table. Participation in the study was independent of medical records and any mental or medical healthcare the service member may have been receiving. Inclusion criteria included being 18 years of age or older, fluent in English, and from a uniformed service (Air Force, Army, Army National Guard, Army Reserves, Coast Guard, Marines, Navy, and U.S. Public Health Service). Exclusion criteria included suicidal or homicidal behavior in the past three months or diagnosis of or care for a psychotic disorder in the past year. Individuals meeting these criteria were administered a screening questionnaire to assess for common symptoms after a “stressful experience” using an aggregate of 26 symptoms of PTSD, depression, and generalized anxiety. PTSD symptoms were measured by 18 of 20 items from the PTSD Checklist for the DSM-5 (PCL-5) [20]. Depression symptoms were measured by 6 of 9 items from the Patient Health Questionnaire Depression Scale (PHQ-9) [21, 22]. Generalized anxiety symptoms were measured by 2 of 7 items from the Generalized Anxiety Disorder-7 (GAD-7) [23]. The response format of the 26 items was modified to an 11-point scale, 0 (*not at all*) to 10 (*extremely*), (range 0–260) and the timing was “…over the past month.” Individuals with a score of 40 or more were enrolled in the study.

#### Assessment of PTSD

Once enrolled, participants completed a pre-questionnaire that included the 20-item PCL-5 [20] to determine DSM-5 probable PTSD versus no PTSD. Response choices were 0 (*not at all*) to 4 (*extremely*). The total symptom severity score range was 0–80. A probable PTSD diagnosis was made by treating each item rated as 2 (*moderately*) or higher as an endorsed symptom, then following the diagnostic criteria requiring a cluster A traumatic exposure (all participants reported exposure to at least one traumatic event), at least 1 cluster B item, 1 C item, 2 D items, and 2 E items, and a symptom severity score of 38 or higher [20]. Participants meeting these criteria were the probable PTSD group (*n* = 42, 52.5%, hereafter referred to as those with PTSD) and those not meeting these criteria were the without PTSD group (*n* = 38, 47.5%). Participants were instructed on completing the “daily diary” (DD) assessments of PTSS and selected the hour of the first daily diary assessment (DD1) that best fit their daily routine. Participants were told to go about their normal daily activities during the DD assessment period and complete assessments at the specified times. DD assessments were scheduled to start the next day.

#### Daily assessments

For the following 15 days, participants completed four DD assessments per day using an EMA methodology. The first 40 consecutive subjects (50.0%) completed DD assessments on paper questionnaires, and the next 40 subjects (50.0%) completed assessments using the same questions on a portable electronic device (Apple Inc. iPad 2 with software designed specifically for use in this study). The phase of the study (paper vs. electronic) was controlled for all analyses and was not a significant covariate. Assessments were at fixed intervals, four hours apart (i.e., if the participant elected to start DD1 at 8 a.m., then DD2, DD3, and DD4 were at 12 p.m., 4 p.m., and 8 p.m., respectively) similar to time block designs in other studies [16]. Participants were instructed to complete assessments within the first two hours when possible, but assessments were accepted for an additional four hours. All participants were provided the options of assessment alerts by text message, voice message, and a 3″ × 2″ × 1″ portable alarm clock capable of 12 programmable alarms per day. Participants completing electronic assessments had the additional option of using alerts that were built into the electronic application. Participants using paper questionnaires recorded the date and time when each assessment was completed, and electronic assessments were automatically date and time stamped. A total of *N* = 3842 assessments were collected. Of those, *n* = 162 (4.2%) paper assessments were not usable because they were completed too early (*n* = 36), too late (*n* = 48), or were missing the completion date or time (*n* = 78). Assessments not completed within the specified six hour assessment period were dropped from data analysis. Of the *N* = 3680 assessments included in the analyses, *n* = 2785 (75.7%) were completed within 0–2 h, *n* = 689 (18.7%) within 2–4 h, and *n* = 206 (5.6%) within 4–6 h.

##### PTSS

Daily PTSS were assessed using 18 of 20 PCL-5 items. The 2 excluded PCL-5 items related to sleep, and therefore were not appropriate for repeated assessment throughout the day. Items were modified to be relevant for repeated assessments as has been done in other research [19]. For example, items in DD1 contained the timing phrase “…since you awakened” and items in DD2, DD3, and DD4 contained the phrase “…in the last couple of hours.” The response format of the 18 items was modified to an 11-point scale, 0 (*not at all*) to 10 (*extremely*), for a total symptom severity score range of 0–180. Research on making these types of response format modifications indicates that changing a 5-point scale to an 11-point scale is unlikely to affect the mean but is likely to produce data with more variance [24]. The 18 items were included on all four DD assessments.

##### Day of week

A 7-day and a dichotomous weekday (Monday through Friday) / weekend (Saturday and Sunday) variable were created to test if the outcome differed by the day of the week. Because we did not find a developmental trend by time in PTSS, a general variable for time was not included in the analyses.

##### Sleep medication, caffeine, and alcohol use

Sleep medication, caffeine, and alcohol use were assessed daily. Sleep medication use was measured in DD1 with one item which asked if participants took any medication (prescribed or over the counter) to help them sleep the previous night. In DD4, caffeine use was measured with two items and alcohol use was measured with one item adapted from Kessler & Ustun [25]. Participants reported how many (1) energy drinks and/or pills, (2) other caffeinated beverages, and (3) alcoholic drinks were used throughout the day. Responses to the two caffeine items were summed for analysis. A binary variable was created on alcohol use due to low rates of use.

### Data analyses

Day of week variation in PTSS was assessed using linear mixed models with DD assessments (level-1) nested within subjects (level-2). To account for unequal time intervals, a spatial power covariance structure was specified. The first-order autoregression assumption, AR(1), was used as it improved model fit compared to compound symmetry. The sample size (*n* = 80) may limit detection of between-subject differences. However, importantly there were *n* = 3680 assessments for the within-subject examination of day of week variation in PTSS. For example, the power to detect a weekday vs. weekend difference among individuals with PTSD was above .95 based on a post hoc power analysis [26]. Analyses consisted of three steps. The first step examined whether participants with and without PTSD differed in PTSS across the seven days of the week or between weekdays and weekends. Day of week, PTSD group, and their interaction term were included in the model. If the interaction term was statistically significant, we conducted the analyses within each PTSD group. Gender, age, race, education, and phase of the study (paper vs. electronic) were included as covariates in all analyses. The Tukey-Kramer method was used to adjust for multiple pairwise comparisons. The third step was to include medication, caffeine, and alcohol use in three separate analyses to examine whether the day of the week variation in PTSS remain the same as in the previous step. All analyses were conducted in PC SAS version 9.3 (SAS Institute, Cary, North Carolina).

## Results

### Sample characteristics

Sample characteristics and descriptive statistics are shown in Table 1. Mean age of participants in the analytic sample (*n* = 80) was 37.2 (range 19–69). Approximately half (56.3%, *n* = 45) were male, mostly Caucasian (73.8%, *n* = 59), and 43.8% (*n* = 35) had some college or technical school education or higher. The majority (57.5%, *n* = 46) were married with 78.3% (*n* = 36) currently living with their spouse. In total, 42 (52.5%) had probable PTSD and 38 (47.5%) did not have PTSD. Mean PTSS scores (range 0–180) were 47.3 (*SD* = 36.8) for the entire sample, 70.6 (*SD* = 33.7) for participants with PTSD, and 21.6 (*SD* = 18.3) for participants without PTSD. On average, sleep medication was used on 39.2% (with PTSD: 46.2%; without PTSD: 31.4%) of days and alcohol was used on 23.3% (with PTSD: 27.3%; without PTSD: 18.8%) of days. The average number of caffeinated beverages and/or pills used per day was 1.38 (with PTSD: 1.51 without PTSD: 1.24). There was no difference between those with and without PTSD in demographic characteristics or sleep medication, caffeine, or alcohol use.

**Table 1:** Demographic Characteristics and Descriptive Statistics of Participants with and without Probable PTSD

	Total (*N* = 80)	Probable PTSD (*N* = 42)	Without PTSD (*N* = 38)
Categorical	*n* (%)	*n* (%)	*n* (%)
Gender			
Male	45 (56.3)	25 (59.5)	20 (52.6)
Female	35 (43.8)	17 (40.5)	18 (47.4)
Race			
Caucasian	59 (73.8)	32 (76.2)	27 (71.1)
Others	21 (26.3)	10 (23.8)	11 (29.0)
Education			
High school or G.E.D	4 (5.0)	2 (4.8)	2 (5.3)
Some college/technical school	41 (51.3)	24 (57.1)	17 (44.7)
Bachelors or Graduate degree	14 (17.5)	6 (14.3)	8 (21.1)
Graduate degree	21 (26.3)	10 (23.8)	11 (29.0)
Marital Status			
Currently married	46 (57.5)	23 (54.8)	23 (60.5)
Not currently married	34 (42.5)	19 (45.3)	15 (39.5)
Living with spouse (among married)			
Yes	36 (78.3)	17 (73.9)	19 (82.6)
No	10 (21.7)	6 (26.1)	4 (17.4)
Continuous	*M* (*SD*)	*M* (*SD*)	*M* (*SD*)
Age	37.2 (10.5)	36.1 (8.8)	38.4 (12.0)
PTSS	47.3 (36.8)	70.6 (33.7)	21.6 (18.3)
Hours of sleep	5.6 (1.4)	5.2 (1.2)	6.0 (1.5)
Sleep medication use, % of days	39.2 (41.1)	46.2 (42.7)	31.4 (38.2)
Caffeinated beverages and/or pills	1.4 (1.2)	1.5 (1.2)	1.2 (1.3)
Alcoholic drinks, % of days	23.3 (29.9)	27.3 (31.7)	18.8 (27.7)

Note. There was no difference between those with probable PTSD and without PTSD in demographic characteristics or sleep medication, caffeine, or alcohol use

### PTSS: Variation by the day of the week

We first examined broadly whether participants with and without PTSD differed in PTSS across the day of the week using mixed models. The interaction of the seven-category day of week variable with PTSD group was not significant, *F*(6, 443) = 1.44, *p =* .197. However, when the dichotomous variable of weekdays and weekends was used, participants with and without PTSD were statistically different in the comparison between weekdays and weekends, *F*(1, 74) = 6.55, *p* = .013. PTSS did not differ significantly by demographic characteristics or by the phase of the study.

**Fig. 1:**
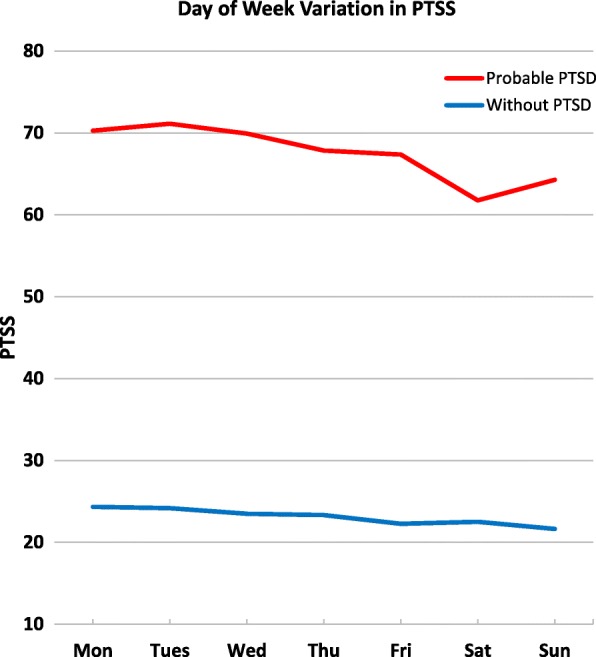
Day of Week Difference in PTSS in Participants with and without Probable PTSD. Note. Least squares means were obtained by estimating PTSS adjusting for demographic characteristics, including gender, age, race, and education. PTSS varied across the seven days among participants with probable PTSD, *F*(6, 228) = 3.05, *p* = .007, but not among those without PTSD, *F*(6, 215) = 0.82, *p =* .559

In order to explore the interaction, we stratified by PTSD group. Results of mixed models are reported in Table 2 and least square means of PTSS by day of week are shown in Fig. 1. Among participants with PTSD, PTSS varied across the seven days *F*(6, 228) = 3.05, *p* = .007, but not among those without PTSD, *F*(6, 215) = 0.82, *p =* .559. Among participants with PTSD, PTSS on Saturday (set as reference for day of week comparison) were lower than any of the other days except for Sunday. Among those without PTSD, PTSS for Saturday were not different from any other day (Table 1).

**Table 2:** Day of Week Difference in PTSS in Participants with and without Probable PTSD: Mixed Models

	Probable PTSD (*N* = 42)		Without PTSD (*N* = 38)	
Parameter	Coefficient [95% CI]	*p*	Coefficient [95% CI]	*p*
Fixed effects				
Intercept	67.37 [44.67, 90.06]	<.001	12.35 [−1.35, 26.04]	.076
Female vs. male	0.90 [−20.45, 22.24]	.933	1.85 [−9.95, 13.65]	.752
Age (centered at 37.2)	−0.02 [−1.71, 1.68]	.985	0.45 [−0.07, 0.97]	.086
Non-White vs. White	−12.46 [−37.65, 12.72]	.322	8.78 [−5.34, 22.90]	.215
Lower than college vs. some college/tech school or higher^a^	−2.51 [− 32.97, 27.94]	.868	3.76 [−9.03, 16.54]	.554
Phase 1 vs. 2	2.86 [−18.16, 23.87]	.784	5.03 [−7.19, 17.25]	.408
Day of Week^b^				
Monday	8.53 [3.19, 13.86]	.002	1.83 [−1.19, 4.85]	.234
Tuesday	9.35 [4.03, 14.68]	.001	1.67 [−1.32, 4.66]	.272
Wednesday	8.15 [2.93, 13.37]	.002	0.98 [−2.04, 4.00]	.523
Thursday	6.11 [0.95, 11.26]	.021	0.83 [−2.12, 3.78]	.580
Friday	5.60 [0.48, 10.73]	.032	−0.23 [−3.12, 2.65]	.873
Sunday	2.51 [−2.76, 7.78]	.348	−0.88 [− 3.84, 2.08]	.560
Random effects				
Between-person variance	1050.46	<.001	270.22	.001
Autocorrelation	0.45	<.001	0.48	<.001
Within-person variance	535.28	<.001	169.12	<.001
Intraclass Correlation^c^	0.66		0.62	

Note. ^a^Some college/technical school or higher was set as the reference. ^b^Saturday was set as the reference. PTSS varied across the seven days among participants with probable PTSD, *F*(6, 228) = 3.05, *p =* .007, but not among those without PTSD, *F*(6, 215) = 0.82, *p =* .559. ^c^Intraclass Correlation was calculated by the ratio of between-person variance and the total variance

To compare between any two days, all pairwise comparisons were conducted. The Tukey-Kramer method was used to adjust for multiple comparisons. Among participants with PTSD, Saturday PTSS were lower than Monday ($\widehat{\beta\ }$= 8.53, *t* = 3.15, *p* = .030), Tuesday ($\widehat{\beta\ }$= 9.35, *t* = 3.46, *p* = .011), and Wednesday ($\widehat{\beta\ }$= 8.15, *t* = 3.08, *p* = .038). Among those without PTSD, none of the comparisons were significant.

**Fig. 2:**
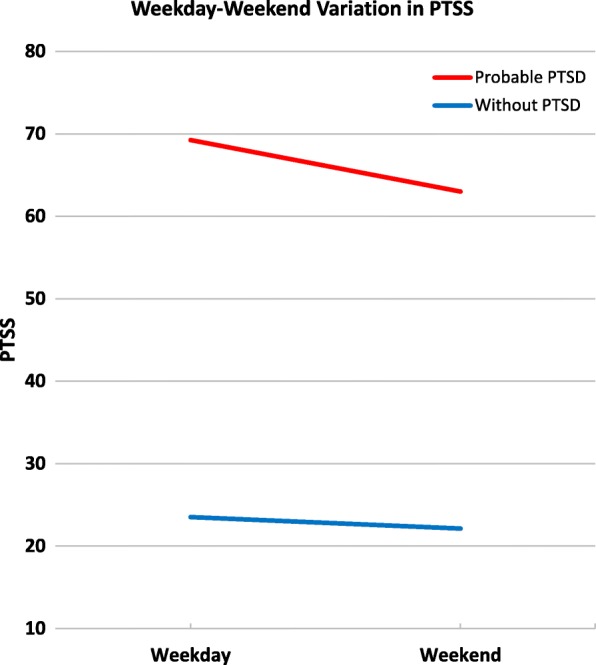
Weekday-Weekend Difference in PTSS in Participants with and without Probable PTSD. Note. Least squares means were obtained by estimating PTSS adjusting for demographic characteristics, including gender, age, race, and education. PTSS was different between weekdays and weekends among participants with probable PTSD, *F*(1, 38) = 14.5, *p* = .001, but not among those without PTSD, *F*(1, 36) = 2.23, *p =* .144

Next, we compared weekdays (Monday through Friday) versus weekends (Saturday and Sunday) to focus on potential differences between traditional work and non-work days. PTSS were higher during weekdays than on weekends among participants with PTSD, $\widehat{\beta}$= 6.24, *F*(1, 38) = 14.5, *p =* .001, but not different among those without PTSD, $\widehat{\beta}$= 1.39, *F*(1, 36) = 2.23, *p =* .144 (Fig. 2).

Among participants with PTSD, the weekday versus weekend remained significant after adjusting in separate models for sleep medication, $\widehat{\beta}$= 6.21, *F*(1, 37) = 14.4, *p =* .001, caffeine, $\widehat{\beta}$= 6.56, *F*(1, 34) = 14.1, *p =* .001, and alcohol use, $\widehat{\beta}$= 6.37, *F*(1, 34) = 132, *p =* .001. Sleep medication ($\widehat{\beta}$= 4.23, *F*(1, 16) = 2.47, *p =* .136), caffeine ($\widehat{\beta}$= − 1.43, *F*(1, 1654) = 3.40, *p =* .065), and alcohol use ($\widehat{\beta}$= − 0.11, *F*(1, 24) = 0.00, *p =* .963) were not significantly associated with PTSS. Similarly, the seven-category day of week remained significant after adjusting for sleep medication, caffeine, and alcohol use.

## Discussion

Using an EMA approach, results of the present study indicated that PTSS varied by the day of the week among those with probable PTSD, but not among those without PTSD. This was true when we examined both across the 7-days of a week and specifically for weekday versus weekend comparisons.

The variation in PTSS was independent of the use of sleep medication, caffeine, and alcohol. In our sample, sleep medication was used an average of 39.2% of days, and rates of caffeine use were 1.38 beverages and/or pills per day on average. There is evidence that PTSD symptoms increase with alcohol consumption [16]. However, reported alcohol use (alcohol consumed on 23.3% of days on average) was low in our sample, and also could not account for these results. The source of the day to day variability in PTSS may be related to other lifestyle and psychosocial factors such as change in sleep [27, 28], leisure activities such as yoga or meditation [29], exercise [30], and social interactions [31]. All of these factors would be expected to differ between weekdays and the weekend. It is also possible that there are day to day differences in factors that may trigger PSTD symptoms. For example, being in the military environment on work days may increase exposures to trauma reminders such as uniforms, equipment, weapons, training exercises, injured soldiers, and combat-related sights, sounds, and experiences that may be triggering factors for PTSD symptoms in our study subjects, and that may not be present in the non-work day environment. Further research is needed to determine the factors that may account for day to day variability in PTSS. Identifying these precipitating and potentially protective factors in the daily lives of individuals with PTSD may suggest environmental and lifestyle interventions to lessen day-to day PTSD symptoms. Future studies should explore additional factors including hours of sleep, traumatic event cues, timing of interpersonal conflicts, and stressful events at work or home [32].

There are several limitations of the present study. Probable PTSD, daily PTSS, and the reported completion time of paper assessments were obtained by self-report. Subjects were recruited at a major military medical center rather than from the community, which may limit the generalizability of our findings. However, there is no evident reason to suspect that this sample was atypical of military service members with PTSD. Since we did not track participants’ work schedule, we do not know to what extent the trends in PTSS vary with work. Study measures of sleep medication, caffeine, and alcohol use were based on self-report, and use of these substances might not have been reported correctly by our subjects. Participants were instructed to take their medications as usual, and to the extent they complied with this request, we would not expect that to influence PTSS variation. However, variation in medication adherence was not examined in the study. Future studies should include objective measures of sleep medication, sleep duration, and caffeine and alcohol use.

The daily assessment and monitoring of PTSS may have implications for the diagnosis of PTSD as well as clinical care and treatment. Currently, the diagnosis of PTSD is based on a single assessment. Daily monitoring of PTSS, like ambulatory monitoring of blood pressure in the evaluation of cardiovascular disease, may reveal diagnostic information about symptom number, severity, and duration that is not evident in a single assessment. Monitoring changes in PTSS across time may lead to a better understanding of individual symptoms and interventions to improve them. By identifying specific psychological or environmental events that precede symptom changes or that may make PTSD symptoms less likely may identify targets for psychotherapeutic intervention. Treatments may be more critical on certain days for patients whose symptoms are likely to increase due to lifestyle or psychosocial factors or potential exposure to trauma reminders. Further study of the day of the week variation in PTSS and other behavioral and lifestyle factors may add to the understanding of PTSD and to identifying modifiable precipitating factors.

## Conclusions

PTSS vary by the day of the week among individuals with PTSD, but not among those without PTSD. This was true across the 7-days of the week and specifically in weekday versus weekend comparisons; there are more symptoms on weekdays compared to weekends. Variation in PTSS was independent of the use of sleep medications, caffeine, and alcohol. Further research is needed to determine the factors that account for daily variation in PTSS. Identifying precipitating and potentially protective factors may inform development of treatments for PTSD.

## Abbreviations

AR(1): Autoregression assumption
DD: Daily diary
EMA: Ecological momentary assessment
GAD-7: Generalized Anxiety Disorder 7
PCL-5: PTSD Checklist for the DSM-5
PHQ-9: Patient Health Questionnaire Depression Scale
PTSD: Post traumatic stress disorder
PTSS: Post traumatic stress symptoms
